# Brain-Derived Neurotrophic Factor Ameliorates Learning Deficits in a Rat Model of Alzheimer's Disease Induced by Aβ1-42

**DOI:** 10.1371/journal.pone.0122415

**Published:** 2015-04-07

**Authors:** Lu Zhang, Yu Fang, Yajun Lian, Yuan Chen, Tianwen Wu, Yake Zheng, Huili Zong, Limin Sun, Ruifang Zhang, Zhenhua Wang, Yuming Xu

**Affiliations:** 1 Key-Disciplines Laboratory Clinical-Medicine of Henan, Zhengzhou, Henan, China; 2 Department of Neurology, First Affiliated Hospital of Zhengzhou University, Zhengzhou, Henan, China; 3 Department of Intensive Care Unit, First Affiliated Hospital, Zhengzhou University Zhengzhou, Henan, China; Institut National de la Santé et de la Recherche Médicale (INSERM U901), FRANCE

## Abstract

An emerging body of data suggests that the early onset of Alzheimer’s disease (AD) is associated with decreased brain-derived neurotrophic factor (BDNF). Because BDNF plays a critical role in the regulation of high-frequency synaptic transmission and long-term potentiation in the hippocampus, the up-regulation of BDNF may rescue cognitive impairments and learning deficits in AD. In the present study, we investigated the effects of hippocampal BDNF in a rat model of AD produced by a ventricle injection of amyloid-β1-42 (Aβ1-42). We found that a ventricle injection of Aβ1-42 caused learning deficits in rats subjected to the Morris water maze and decreased BDNF expression in the hippocampus. Chronic intra-hippocampal BDNF administration rescued learning deficits in the water maze, whereas infusions of NGF and NT-3 did not influence the behavioral performance of rats injected with Aβ1-42. Furthermore, the BDNF-related improvement in learning was ERK-dependent because the inhibition of ERK, but not JNK or p38, blocked the effects of BDNF on cognitive improvement in rats injected with Aβ1-42. Together, our data suggest that the up-regulation of BDNF in the hippocampus via activation of the ERK signaling pathway can ameliorate Aβ1-42-induced learning deficits, thus identifying a novel pathway through which BDNF protects against AD-related cognitive impairments. The results of this research may shed light on a feasible therapeutic approach to control the progression of AD.

## Introduction

Alzheimer's disease (AD) is the most common cause of dementia worldwide and currently affects more than 6% of the population over the age of 65 [1,2]. Among the most robust pathologies observed in AD are the accumulation and deposition of senile plaques composed of amyloid-β peptides (Aβ) in the brain. Specifically, Aβ is derived from amyloid precursor protein (APP) by β- and γ-secretase, producing 40–42 amino acid Aβ peptides; these plaques can cause inflammatory responses and neuronal cell death [3]. The neurite atrophy and synaptic loss induced by Aβ are considered to be the major causes of gradual cognitive deterioration in AD sufferers [4,5]. Therefore, investigating treatments that target amyloid-based molecular mechanisms is a rational strategy for developing novel therapeutics to treat this disorder.

Accumulating evidence suggests that brain-derived neurotrophic factor (BDNF) is implicated in the pathophysiology of various CNS diseases (for a review see [6]). BDNF is a small (27 kDa) dimeric protein structurally related to nerve growth factor (NGF) and widely expressed in the mammalian brain [7]. BDNF plays an important role in the growth, development, differentiation, maintenance and regeneration of various types of neurons in the CNS [8,9]. As a neurotransmitter modulator, BDNF participates in plasticity processes, such as long-term potentiation [10] and long-term depression [11,12], as well as learning and memory [13–16].

Previous studies have shown that a reduced expression of BDNF mRNA and protein is found in specific brain regions of postmortem AD samples, especially in the hippocampus [17,18]. Specifically, using immunohistochemical staining, Conner and colleagues found a reduction in the intensity and number of BDNF-immunoreactive cell bodies in both the hippocampus and temporal cortex in AD samples [19]. The basal forebrain cholinergic neurons of AD patients exhibit a reduced level of BDNF protein, as well [20]. The catalytic form of the surface receptor of BDNF, tyrosine receptor kinase B (TrkB), is decreased in the hippocampal formation in AD patients, whereas its truncated form is increased [21]. Using real time (RT)-PCR, Fahnestock and colleagues have demonstrated that BDNF is synthesized in the basal forebrain and supplies cholinergic neurons with a local and target-derived source of this factor [20]. Other studies have noted that this neurotrophin promotes the survival of all major neuronal types affected in AD, including hippocampal, neocortical, cholinergic septal, and basal forebrain neurons [22]. In addition, Aβ-associated neurotoxicity and dendrite atrophy may be a consequence of BDNF deficiency. Both extensive amyloid pathology and decreased BDNF levels were noted in the cortex and hippocampus of individuals with AD (for a review see [6,23]).

Implicit memory loss is the earliest and most frequently reported symptom often preceding the onset of clinical dementia [24]. BDNF is highly implicated in spatial learning and memory via cAMP response element binding protein (CREB)- and extracellular signal-regulated protein kinase (ERK)-dependent mechanisms during hippocampal LTP, a widely studied synaptic model of memory [25,26]. BDNF most likely protects against AD by promoting neuronal survival and facilitating the activity-dependent plasticity that underlies the capacity for learning and memory. To date, however, the effect of BDNF on Aβ-induced learning deficits remains elusive. In this study, we find that a ventricular injection of Aβ1–42 significantly impairs spatial learning and memory in the Morris water maze (MWM), and this learning deficit is correlated with a decrease in hippocampal BDNF levels. Furthermore, the infusion of BDNF in the hippocampus reverses the learning and memory impairment observed in the Aβ1-42-treated rats, and the beneficial effect of BDNF on memory is dependent on the ERK pathway.

## Material and Methods

### Main reagents and drugs

Amyloid-β1–42 (Aβ1–42) was obtained from Sigma-Aldrich (St. Louis, MO, USA). Before injection, the Aβ1–42 peptide was dissolved in a physiological saline solution at a concentration of 5 mg/ml and incubated at 37°C for 72 h to induce aggregation. Human full length BDNF (ab9794), NGF (ab138794) and NT-3 (ab138798) proteins and their corresponding rabbit polyclonal antibodies (ab75040, ab6198 and ab65804) were purchased from Abcam (Cambridge, UK). Rabbit monoclonal antibodies against phospho-ERK (#4370), total ERK (#4695), phospho-JNK (#4668), total JNK (#9258), phospho-p38 (#4511), total p38 (#9212) and GAPDH (#5174) were from Cell Signaling Technology (Beverly, MA, USA). Ceramide C6, an ERK activator, was obtained from Santa Cruz Technology (Santa Cruz, CA, USA). The ERK inhibitor PD98059 was from Sigma-Aldrich (St. Louis, MO, USA).

### Animals

A total of 118 healthy male Sprague-Dawley rats (Experimental Animal Center of China Medical University, Shenyang, China), ranging in age from 7 to 8 weeks (weight 200±18 g) at the beginning of the experiment, were housed in a room maintained at 23°C with a 12-hour light-dark cycle. The rats were given free access to food and water except during the behavioral test. The present study were approved by the Animal Care and Use Committee of Zhengzhou University and performed in accordance with the National Institutes of Health Guidelines for the care and use of laboratory animals. Every efforts were made to minimize suffering of the animals.

### Surgery and induction of Alzheimer's disease

The animal model of Alzheimer's disease was prepared using the Aβ1–42 aggregates intracerebroventricular (i.c.v.) injection method. The rats were anesthetized with an intraperitoneal (i.p.) injection of chloral hydrate (350 mg/kg) and mounted on a stereotaxic apparatus (RWD Life Science Co., Ltd, Shenzhen, China). The skulls of the rats were opened and drilled with burr holes on both the sides of the corresponding position to allow i.c.v. injection of Aβ1–42 (anteroposterior: -0.8 mm from Bregma, medial/lateral: ±1.4 mm and dorsal/ventral: -4.0 mm) or intra-hippocampal injection with other drugs (anteroposterior: -3.5 mm from Bregma, medial/lateral: ±2.0 mm and dorsal/ventral: -2.8 mm). For the injection of Aβ1–42, two small holes were made and Aβ1–42 (2.0 μl per side) was injected bilaterally into the lateral ventricles through a stainless steel cannula using a Hamilton microsyringe. The injection lasted 5 min, and the needle with the syringe was left in place for 2 min after the injection to ensure complete infusion of the drug. Sham rats were injected with the same volume of saline as control of Aβ1-42-injected rats. After surgery, two stainless steel obturators were inserted into the guides to prevent cannula occlusion. After surgery, the rats were housed individually and had access to food and water freely. Penicillin was applied daily, and the rats were allowed 5 days to recovery from surgery. No unintended deaths of animals during the surgery. The general condition of the animals, including the body weight, food and water intake, was monitored daily after surgery. Six rats were excluded due to obvious movement defects during the recovery period. All rats were then randomly assigned to different treatment groups.

For intra-hippocampal injection, two guide cannulae (21-gauge) were inserted into the hippocampus and anchored to the skull with sterile stainless steel screws and acrylic dental cement. BDNF (0.05, 0.25 and 1.0 μg/side), NGF (0.25 μg/side), NT-3 (0.25 μg/side) or the same volume of 0.9% saline was infused bilaterally (in a total volume of 1.0 μl/side) into the hippocampus through the guide cannulae when needed.

### Morris water maze task

**Fig 1:**
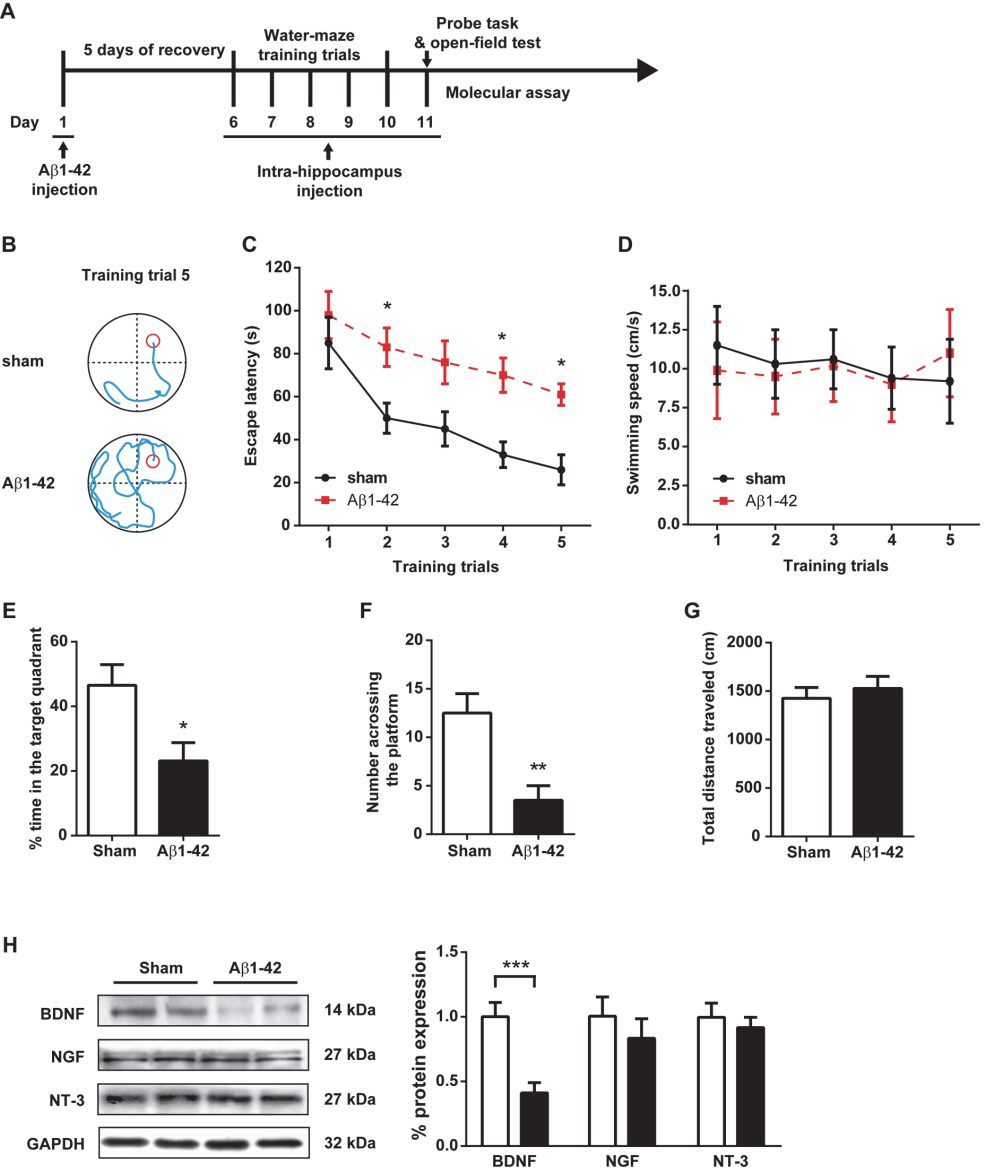
Effect of intracerebroventricular injection of Aβ1–42 aggregates on spatial learning memory in the Morris water-maze test. (A) The experimental protocol. (B) Representative swim traces of each group in the fifth training trial. (C) Escape latency and (D) swimming speed in each training trial were also analyzed. (E) The time spent in the target quadrant in the probe task. (F) The number of times crossing the platform in the probe task. (G) The total distance traveled in the open field test after the probe task. (H) The expression of BDNF, NGF and NT-3 protein in hippocampus in the sham and Aβ1–42 aggregate-treated rats. n = 8 each group. * *P* < 0.05 and ** *P* < 0.01 compared with sham controls.

Spatial learning memory was assessed with the Morris water maze. The experimental apparatus (RWD Life Science, Shenzhen, China) consisted of a circular water pool (diameter 150 cm; height 60 cm; containing water at 24±2°C) divided into four equally spaced quadrants. The pool was placed in a test room containing various prominent visual cues. A translucent 10×10 cm platform, submerged 1 cm below the water surface, was hidden in the center of quadrant II during the training period and was then removed at the time of the probe task. Memory training was performed 5 days after Aβ1–42 injection. The training was conducted twice a day for five consecutive days before the probe task. Each rat was allowed to swim until it found the platform or until 120 s elapsed. Then, the rat was left on the platform for 10 s. During the spatial probe task, the platform was removed from the pool and the rats were allowed to swim for 120 s. The swim escape latency, average swim speed, time spent in the target quadrant, and number of times the animal crossed the previous location of the platform were recorded by a video tracking system (SMART, Panlab SL, Barcelona, Spain). The experimental procedure is outlined in Fig 1A. The performance in the non-target quadrants (ie. quadrant I, III and IV) were also analyzed and presented in the supplemental figure (S1 Fig).

### Open field test

To verify the effects of i.c.v. treatment with Aβ1–42 on locomotor activity, the animals were placed for 10 min in the open field arena after the last Morris water maze test. A black square arena (100 × 100 × 60 cm) was used for the test. Each rat was placed in the center of the arena and was allowed to explore the apparatus freely for 15 minutes with the experimenter out of the animal’s sight. The total distance travelled was analyzed by video-tracking software (SMART, Panlab SL, Barcelona, Spain).

### Western blot

At the end of the final behavioral test, the rats were deeply anesthetized with i.p. chloral hydrate under non-stress conditions and killed by rapid decapitation. The brains were quickly removed and the hippocampal tissues were carefully dissected on ice. To extract the protein, frozen tissues were homogenized in a pre-cooled RIPA buffer (50 mM Tris-HCl, 50 mM NaCl, 5 mM EDTA, 10 mM EGTA, 2 mM sodium pyrophosphate, 4 mM paranitrophenylphosphate, 1 mM sodium orthovanadate, 1 mM phenylmethylsulfonyl fluoride, 2 μg/ml aprotinin, 2 μg/ml leupeptin and 2 μg/ml pepstatin, pH 7.5). The homogenates were incubated on ice for 30 minutes and centrifuged at 12000 × *g* for 15 min at 4°C. The protein content was determined using the bicinchoninic acid method (Joincare Co., Zhuhai, China). The protein samples were subjected to 12% SDS-PAGE and transferred to PVDF membranes. The membranes were blocked for 1 h with 5% fat milk in Tris-buffered saline (500 mM NaCl, 20 mM Tris-HCl, pH 7.5) containing 0.05% Tween-20 and were incubated overnight with primary antibodies at 4°C (all diluted at 1:1000). The next day, the membranes were washed three to four times with 0.1%Tween-20 TBS (pH 7.6) and incubated with horseradish peroxidase-conjugated anti-rabbit or anti-mouse secondary antibodies. An enhanced chemiluminescence kit (Millipore, MA, USA) was used to detect immunoreactive protein bands. The blots were normalized to GAPDH (1:1000).

### Statistical analysis

Statistical analysis was performed using one- or two-way ANOVA followed by Dunnett’s post hoc test, and the results were expressed as the means± SEM. F, DFn, DFd and *P* indicate the value of the F-test, the degrees of freedom of the numerator and denominator and the significance, respectively, and were used to determine whether the factors have significant effects on the result. For the analysis of western blots, the detected bands were calculated densitometrically using QuantityOne (Bio-Rad). The relative protein phosphorylation was expressed as phosphorylated protein *vs* total protein. Significance was accepted at *P* < 0.05.

## Results

### Intracerebroventricular injection with Aβ1–42 markedly attenuates cognitive function and hippocampal BDNF expression in rats

The experimental protocol is shown Fig 1A. Rats were randomly divided into two groups for stereotaxic surgery: a sham-operated group (n = 8) and an Aβ1–42 i.c.v. treatment group (n = 8). The rats in the Aβ1–42 group were administered Aβ1–42 aggregates into the lateral ventricles. The ability of the rats to learn and process spatial information was tested by the Morris water maze. The representative navigation paths at the end of the water-maze training (day 10, training trial 6) demonstrated that spatial learning acquisition was impaired in the rats of the Aβ1–42 group relative to the rats of the sham control group (Fig 1B). The navigation trial analysis showed that the escape latencies decreased from day 1 to day 5 in both the sham and Aβ1-42-treatedgroups [F_training (4, 70)_ = 9.16, *P*< 0.0001] (Fig 1C). However, i.c.v. administration of Aβ1–42 significantly attenuates the spatial learning ability in rats [F_treatment (1, 70)_ = 30.3, *P*< 0.0001]. The post hoc analysis revealed that the Aβ1-42-treated rats showed longer escape latencies than the sham rats at the end of the training (day 10) (*P*< 0.05). We also analyzed the average swimming speed during the water-maze training (Fig 1D). Neither the training [F_training(4, 70)_ = 0.1072, *P* = 0.9796] nor the Aβ1–42 treatment [F_treatment (1, 70)_ = 0.03253, *P* = 0.8574] have significant effects on the swimming speed of rats, indicating that this animal model does not present any motor deficits. In probe trials, rats in the Aβ1–42 group spent less time in the right quadrant (Fig 1E) and fewer attempts at searching for the platform (Fig 1F) compared with the sham controls (*P* < 0.05 and *P* < 0.01, respectively), suggesting the spatial-cognitive ability and memory of the rats was significantly attenuated by i.c.v. injection of Aβ1–42. No obvious behavioral difference was found in the non-target quadrants in two groups (S1A Fig). No difference in the total distance was found in the open field test (*P* = 0.6104) (Fig 1G), also indicating that those rats did not exhibit any alterations in locomotor activity.

Hippocampal BDNF, NGF and NT-3 protein expression was assessed by western blot detection of hippocampal tissues. As shown in Fig 1H, i.c.v. administration of Aβ1–42 suppressed the production of BDNF by 0.43±0.09-fold compared with the sham control group (*P* < 0.0001). In contrast, NGF and NT-3 immunoreactivities were not changed in the hippocampus in the Aβ1–42 group (*P* = 0.4363 and *P* = 0.5658, respectively).

### Administration of BDNF into the hippocampus improves cognitive function in an Aβ1-42-treated rat model of Alzheimer's disease

**Fig 2:**
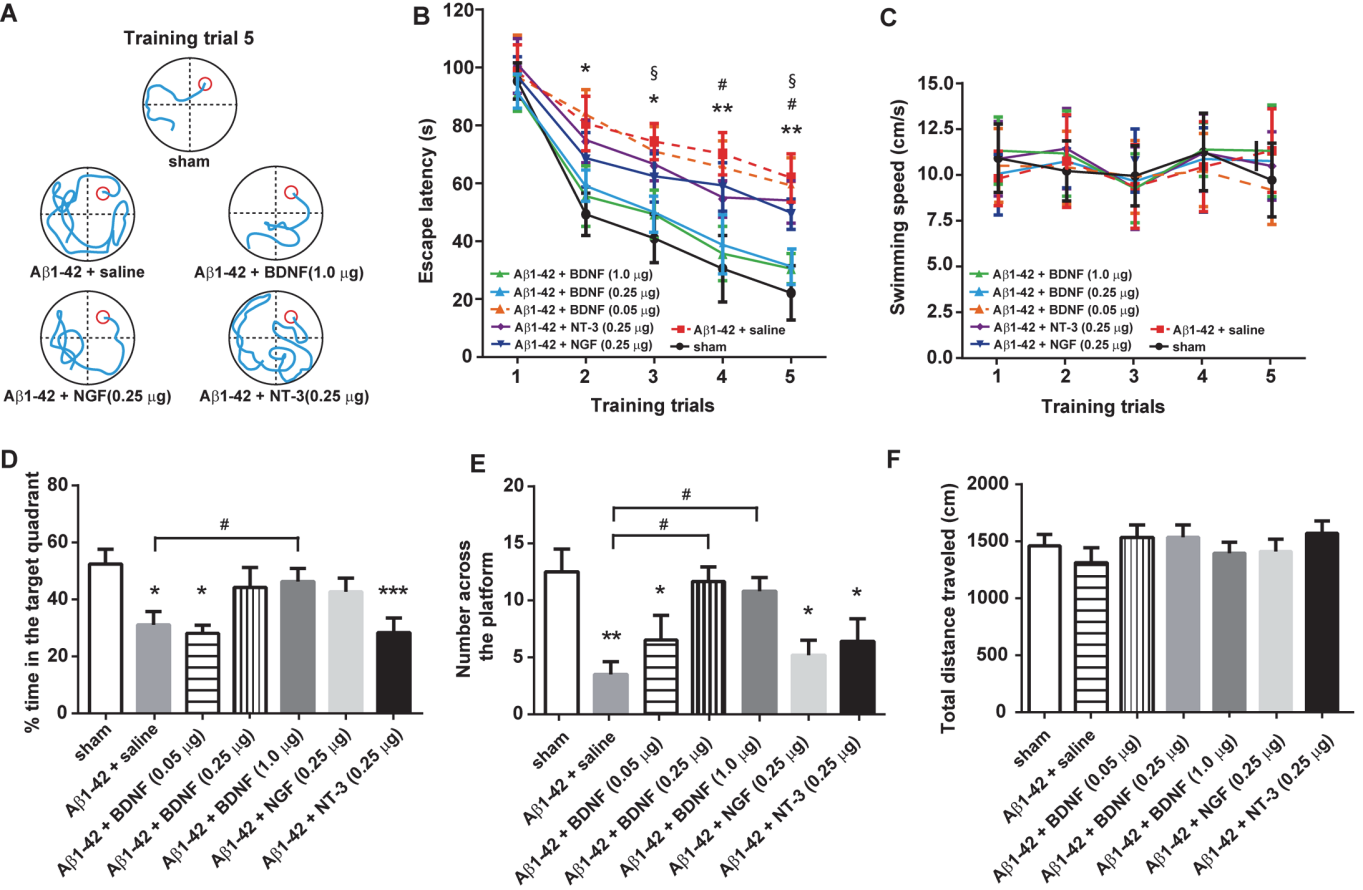
Effect of intra-hippocampal injections of BDNF (0.05, 0.25 and 1.0 μg/side), NGF (0.25 μg/side) and NT-3 (0.25 μg/side) on spatial learning function in the Aβ1-42-treated rat model of Alzheimer's disease. (A) Representative swim traces of each group in the fifth training trial. (B) Escape latency and (C) swimming speed in each training trial were also analyzed. (D) The time spent in the target quadrant in the probe task. (E) The number of times crossing the platform in the probe task. (F) The total distance traveled in the open field test after the probe task. n = 8/group. For panel B, * *P* < 0.05, ** *P* < 0.01, Aβ1–42 + saline *vs* sham control group; # *P* < 0.05, Aβ1–42 + NGF *vs* sham control group; § *P* < 0.05, Aβ1–42 + NT-3 *vs* sham control group. For the other panels, * *P* < 0.05, ** *P* < 0.01, and *** *P* < 0.0001, compared with sham controls. # *P* <0.05 as compared with Aβ1–42 + saline group.

BDNF (0.05, 0.25 and 1.0 μg/side), NGF (0.25 μg/side), NT-3 (0.25 μg/side) or saline (n = 8/group) were injected bilaterally into the hippocampus 30 min before the water-maze training. The representative swimming tracks of rats with different drug treatments after 5 days of water-maze training are shown in Fig 2A. A two-way ANOVA revealed significant effects of intra-hippocampal drug treatments on escape latency [F_treatment (6, 245)_ = 11.65, *P*< 0.0001] (Fig 2B). As expected, the Aβ1–42 + saline-treated rats showed a significant delayed escape latency after 5 days of water-maze training compared with the sham controls (*P* < 0.01). The NT-3- and NGF-treated rats also showed significantly longer escape latencies than those in the sham group (both *P* < 0.05). However, no difference was found between the Aβ1–42 + BDNF (0.25 and 1.0 μg)-treated rats and the sham rats (*P* = 0.8766 and 0.6335, respectively). Neither the training [F_training(4, 245)_ = 0.0561, *P* = 0.9942] nor the treatments [F_treatment (6, 245)_ = 0.3094, *P* = 0.8714] have significant effects on the swimming speed of rats, indicating that those rats do not exhibit any locomotor deficits (Fig 2C). In the probe task, an intra-hippocampus injection of 0.25 and 1.0 μg BDNF and 0.25 μg NT-3 significantly ameliorated the Aβ1-42-induced decreases in both time spent in the right quadrant (Fig 2D) and attempts for searching the platform (Fig 2E). Moreover, a significant difference in the time spent in the right quadrant was found between Aβ1–42 + (0.25 μg) BDNF and Aβ1–42 + saline-treated rats. There were also significant elevations in BDNF (0.25 and 1.0 μg)-treated rats (both *P* < 0.05) when compared with the Aβ1–42 + saline group. No obvious difference was found in the non-target quadrants between different groups in rats (S1B Fig). No difference in the total distance was observed in the open field test [F_(4, 35)_ = 0.5354, *P* = 0.7106] (Fig 2F).

**Fig 3:**
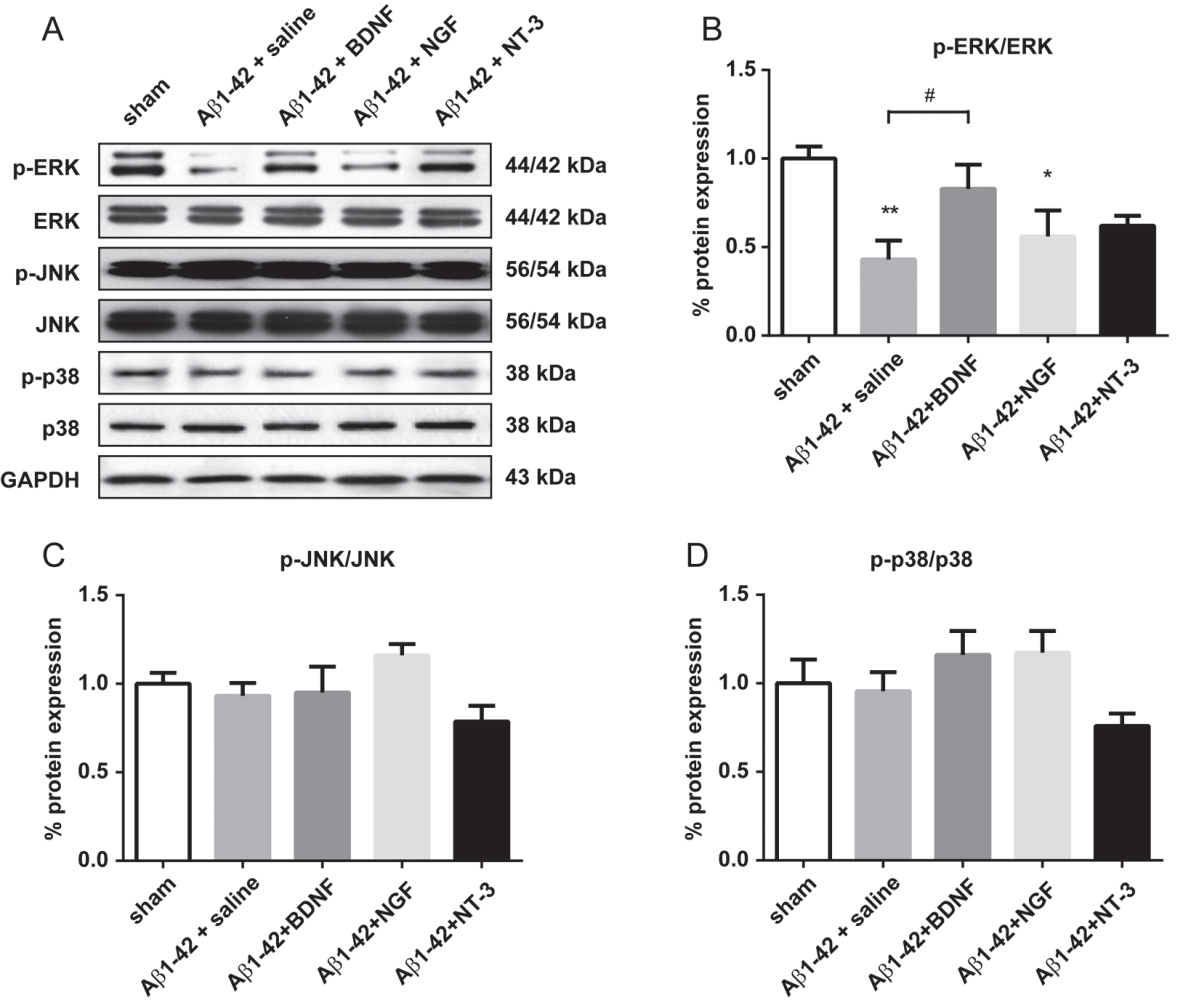
Effect of intra-hippocampal injections of BDNF (1.0 μg), NGF (0.25 μg), and NT-3 (0.25 μg) on MAPK expression in the hippocampus in a rat model of Alzheimer's disease. (A) representative western blots of each group. GAPDH was used as the internal loading control. (B) relative expression of phosphorylated ERK. (C) and (D) relative expression of phosphorylated JNK and p38. * *P* < 0.05, ** *P* < 0.01, and *** *P* < 0.0001 compared with sham controls. # *P* <0.05 as compared with Aβ1–42 + saline group.

To observe the effects of BDNF (both 0.25 and 1.0 μg/side have the same behavioral effects; therefore, rats in the 0.25 μg/side group were chosen for further analysis), NGF (0.25 μg/side) and NT-3 (0.25 μg/side) on MAPK activation in the Aβ1-42-treated rat model of Alzheimer's disease, we analyzed the expression of total ERK, JNK and p38, as well as their phosphorylated (activated) forms, in the hippocampus after treatment (Fig 3). The quantification of ERK (44/42 kDa) and JNK (56/54 kDa) were based on the summation of both bands. A one-way ANOVA revealed a significant effect of different treatments on ERK phosphorylation [F_(4, 35)_ = 4.323, *P* < 0.01] but not on JNK [F _(4, 35)_ = 2.152, *P* = 0.0949] or p38 phosphorylation [F_(4, 35)_ = 2.121, *P* = 0.0989]. Post hoc analysis showed that ERK phosphorylation in the Aβ1–42 + saline (*P* < 0.01) and the Aβ1–42 + NGF group (*P* < 0.05) were significantly lower than the sham control group. A significant difference was found between Aβ1–42 + saline and Aβ1–42 + BDNF rats. Phospho-ERK expression in the BDNF- (*P* = 0.6416) and NT-3-treated (*P* = 0.0613) rats showed no difference compared with the sham controls.

### BDNF improves cognitive function through ERK activation in the hippocampus

**Fig 4:**
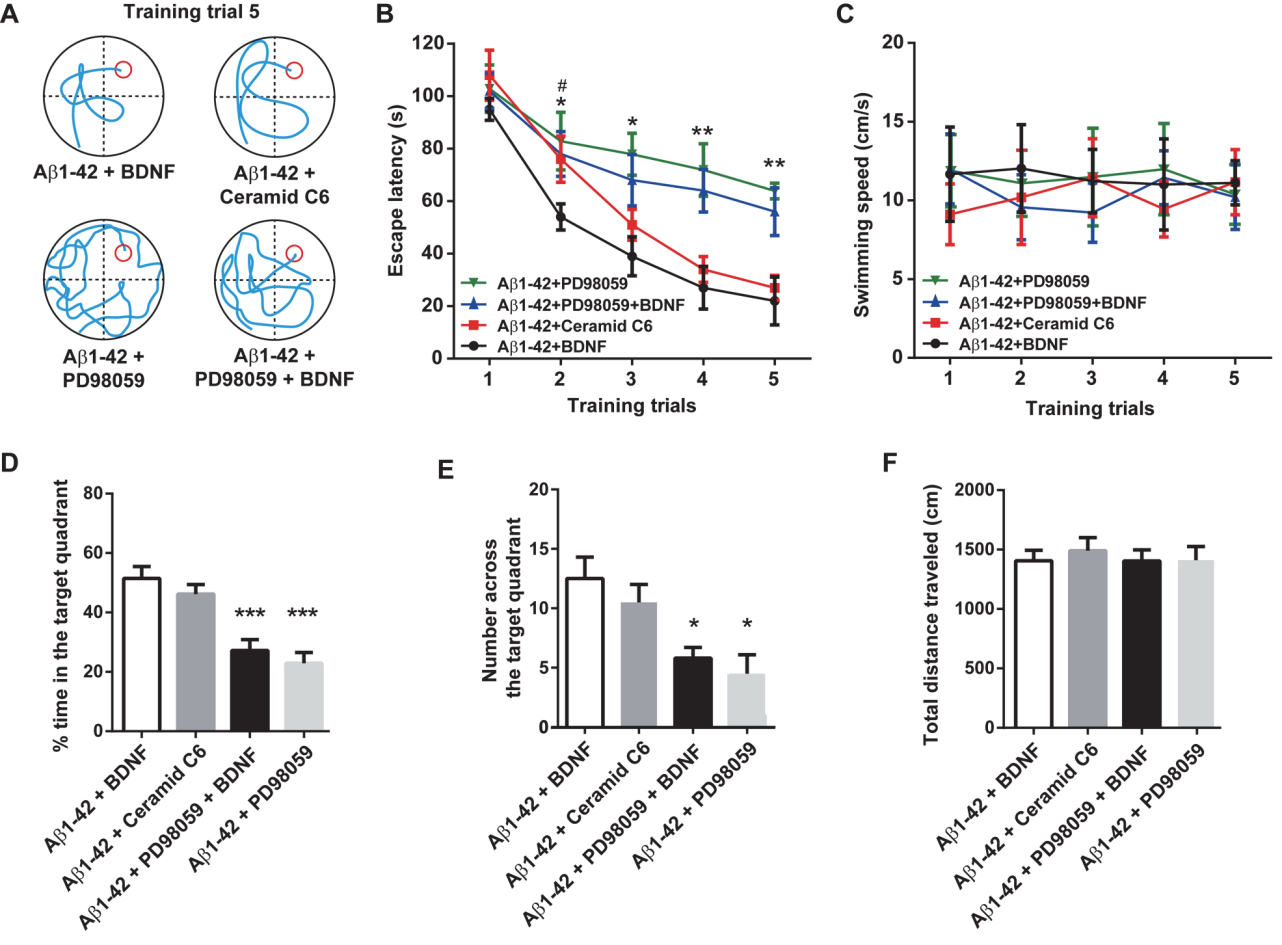
Effect of intra-hippocampal injections of BDNF, ERK activator ceramide C6 and the ERK inhibitor PD98059 on spatial learning function in the rat model of Alzheimer's disease. (A) Representative swim traces of each group in the fifth training trial. (B) Escape latency and (C) swimming speed in each training trial were also analyzed. (D) The time spent in the target quadrant in the probe task. (E) The number of times crossing the platform in the probe task. (F) The total distance traveled in the open field test after the probe task. n = 8 each group. For panel B, * *P* < 0.05, ** *P* < 0.01, Aβ1–42 + PD98059 + BDNF *vs* Aβ1–42 + BDNF group; # *P*< 0.05, Aβ1–42 + ceramide C6 *vs*. Aβ1–42 + BDNF group. For the other panels, * *P* < 0.05 and *** *P* < 0.0001 compared with the Aβ1–42 + BDNF group.

BDNF (1.0 μg, n = 16), the ERK activator ceramide C6 (50 nM, n = 8), the ERK inhibitor PD98059 (20 μM, n = 8) or BDNF combined with PD98059 (20 μM, n = 8) was injected bilaterally into the hippocampus 30 min before each water-maze training trial and probe task. The representative swimming tracks of the rats in the fifth water-maze training trial are shown in the Fig 4A. A two-way ANOVA revealed a significant effect of intra-hippocampal drug treatments on escape latency [F_treatment (3, 180)_ = 14.91, *P*< 0.0001] (Fig 4B). No obvious difference was found between the Aβ1–42 + BDNF group and the Aβ1–42 + ceramide C6 group (*P* = 0.8538) in escape latency in the fifth training trial. However, the escape latency was significantly increased in rats treated with Aβ1–42 + BDNF + PD98059 compared with the Aβ1–42 + BDNF group (*P* > 0.01). Furthermore, no difference was found between the Aβ1–42 + BDNF + PD98059-treated rats and the Aβ1–42 + PD98059-treated rats (*P* = 0.8616). No changes in swimming speed were observed in those rats [F_training(4, 180)_ = 0.0203, *P* = 0.9992] [F_treatment (3, 180)_ = 0.3646, *P* = 0.7786] (Fig 4C). In the probe task, the ceramide C6-treated rats showed a similar time spent in the right quadrant (Fig 2D) and number of times crossing the platform (Fig 2E) compared with the Aβ1–42 + BDNF-treated rats. However, intra-hippocampal administration of the ERK inhibitor PD98059 alone or combine with BDNF treatment both showed significant decreases in the time spent in the right quadrant (both *P* < 0.0001) and the attempts at searching for the platform (both *P* < 0.05), indicating that blocking the activity of ERK can not only induce cognition impairments but also eliminated the curative effect of BDNF in the Aβ1-42-treated rat model of Alzheimer's disease. No obvious difference in time spent in the non-target quadrant was found in different groups (S1C Fig).

## Discussion

Several lines of evidence suggest that Aβ plays a central role in the pathogenesis of AD, but the underlying mechanisms remain uncertain. Previous studies provide support for neurotrophic factors as promising drug targets of AD by showing that BDNF can prevent the death of injured adult neurons in the hippocampal formation, cortex and basal forebrain [27,28]. According to this view, decreased levels of BDNF could contribute to the neurite atrophy and synaptic loss observed in the brains of AD patients, and up-regulation of BDNF could control the progression of AD and cognitive decline [29,30]. However, whether the injury models utilized in previous studies reflect the process of neuropathology and cognitive impairments in AD remains unclear. The results of the present study provide direct support for this neurotrophic factor hypothesis, demonstrating that the infusion of BDNF into the hippocampus reverses the learning deficits in the Aβ1-42-induced AD rat model.

The pathology associated with AD includes the loss of basal forebrain cholinergic neurons and presynaptic terminals in the neocortex and hippocampus and a decrease in the total amount of neuronal nicotinic acetylcholine receptors (nAChRs). Aβ1–42 is thought to be a major mediator of the cognitive deficits in AD. Gil-Bea and colleagues have shown that cholinergic deafferentation of the hippocampus induces memory impairments in the water maze test that are correlated with hippocampal activity-regulated cytoskeleton associated protein (Arc) and BDNF downregulation [31]. Arc, a synaptic activity-induced effector molecule, is required for hippocampal long-term potentiation (LTP) and hippocampus-dependent learning and memory [32]. In the adult hippocampus, BDNF induces LTP correlated with the up-regulation of Arc, and these processes are dependent on the ERK activation coupled with the transcription factor cAMP-response element binding protein (CREB) [33]. Consistent with these findings, our results have indicated that the deficits of spatial memory observed in the Aβ1-42-treated rats can be reversed by intra-hippocampus BDNF infusion, and these effects are dependent on ERK activation. Importantly, although phospholipase C (PLC) γ and phosphatidylinositol 3-kinase (PI3K) have also been implicated in BDNF signaling, their activation is not required for spatial long term memory recovery in AD model rats because neither PLCγ inhibitor U-73122 nor PI3K inhibitor wortmannin blocks the beneficial effect of hippocampal BDNF on long term memory restore in the Aβ1-42-injected rats (data not shown).

The accumulation of pathogenic Aβ assemblies in the AD brain results in the progressive dismantling of synapses, neuronal circuits and networks. Previous studies have shown that elevated Aβ attenuates excitatory synaptic transmission by decreasing the number of surface AMPA receptors (AMPARs) and NMDA receptors (NMDARs), which is in turn associated with a collapse of glutamatergic dendritic spines [34–36]. Our data have indicated that the direct infusion of Aβ1–42 results in a reduced BDNF expression in the hippocampus. Consistently, Garzon and Fahnestock have reported that oligomeric Aβ1–42 can decrease BDNF transcripts IV and V in a human neuroblastoma cell line [37]. This decrease is correlated with the inhibition of phosphorylated CREB by BDNF. The activation of CREB is thought to be one of the key downstream mechanisms by which BDNF promotes neuronal survival [38]. Similarly, Aβ down-regulates the BDNF-induced activation of critical transcription factors, such as CREB and Elk-1, through suppressing the Ras-MAPK/ERK and PI3K/Akt pathways in cultured cortical neurons [39].

One hypothesis suggests that a reduced endogenous BDNF expression alters synaptic plasticity and thereby contributes to cognitive impairment in AD. Indeed, BDNF increases synaptic strength [40] and modifies dendritic spine shape [41], although the direct microinjection of BDNF does not exert beneficial effects on age-related memory deficits [42,43]. Additionally, transgenic mice with over-expression of BDNF exhibit learning deficits. These results suggest that BDNF plays a more important role in certain pathological conditions such as AD. BDNF improved cognitive performance in the water maze test in a rodent model of AD in the present study, suggesting that the disruption of endogenous BDNF signaling by Aβ, the primary insult in AD, impairs mnemonic processes in animals. Notably, our results have shown that selected doses of BDNF administrated (0.25 μg and 1.0μg) which are beneficial to AD-related learning deficits are much higher compared with endogenous BDNF levels (around 0.2 ng per 100,000 cells) under physiological conditions [44,45].

ERK, an enzyme important for long-term neuronal plasticity, is found to be a downstream effector in the BDNF signaling cascade. Inhibition of ERK impairs both the maintenance of LTP and the consolidation of long-term memory [46]. Altogether, sublethal levels of Aβ impair BDNF-mediated gene regulatory activities and may thus underlie the deficits of synaptic plasticity related to AD. Consistent with these results, our results have indicated that the beneficial effect of BDNF on cognitive functions in AD depend on ERK activation because pretreatment with an ERK antagonist completely blocked the protective effect of BDNF in the Aβ1-42-induced AD model. In addition, a recent report by Jo and colleagues have shown that the signaling pathway involving caspase-3, Akt1 and glycogen synthase kinase-3β (GSK3β) is an important mediator in Aβ1-42-dependent inhibition of hippocampal LTP in rodents [47]. The exact role of the AKT-GSK3β signaling pathway in AD-related cognitive impairments requires further investigation.

The protecting effect of neurotrophic factors infusions on cognitive impairments were BDNF-specific. Differential expression of the neurotrophic factor receptors, including TrkA, TrkB and TrkC, in hippocampal sub-regions could at least partially account for the neurotrophic factor specificity. The infusions of NGF were expected to have no effect on cognitive improvement because of low TrkA expression in all subfields of the hippocampus [48]. The lack of effect of NGF provides an excellent control for these studies because it has approximately the same molecular weight and similar structure as BDNF and NT-3. TrkB and its ligand BDNF are expressed at relatively high levels in the dentate gyrus and CA3 pyramidal cell layers, and this could account for its behavioral beneficial effects [48,49]. TrkC and NT-3 are also expressed in the dentate gyrus, whereas NT-3 infusions could not produce a similar behavioral effect as BDNF, suggesting that the high levels of the appropriate neurotrophic factor is not the only factor that determines a behavioral consequence. The subcellular localization, such as localization within the cell body, axon or dendrite, of Trk receptors or the differential expression of the intracellular signaling machinery necessary to respond to receptor activation may account for the observed selectivity. Notably, our data have shown that Aβ-associated neurotoxicity might be a consequence of BDNF deficiency, however the TrkB receptor can also be impaired as recently showed by Jerónimo-Santos and colleagues [50].

In summary, we demonstrate that BDNF is down-regulated in hippocampus of rats after a ventricle injection of Aβ1–42. These AD model rats exhibit obvious cognitive deficits in the water maze task. The memory impairments induced by Aβ1–42 were corrected with an intra-hippocampal infusion of BDNF, whereas NGF and NT-3 did not produce these effects. Furthermore, the beneficial effects of BDNF on cognitive functions were dose-dependent and ERK-dependent. This study has provided direct evidence that BDNF produces a selective but beneficial effect on memory improvement in an AD animal model, which should further establish the use of BDNF as a valid neuroprotective agent for AD-related dementia.

## Supporting Information

S1 Fig Time spent for the non-target quadrants in the probe task of Morris water maze. (A) Performance of rats in the Aβ1–42 and sham group. An schematic diagram showed the position for the four quadrants. (B) Performance of rats after intra-hippocampal injection with BDNF, NGF or NT-3. (C) Swimming time for the BDNF, Ceramid C6, PD98059 intra-hippocampal treated rats.
(TIF)
