# Supplementary figures and images for: Brain-Derived Neurotrophic Factor Ameliorates Learning Deficits in a Rat Model of Alzheimer's Disease Induced by Aβ1-42
Source: PLoS One. 2015 Apr 7;10(4):e0122415. doi: 10.1371/journal.pone.0122415 (PMC4388634; doi:10.1371/journal.pone.0122415)

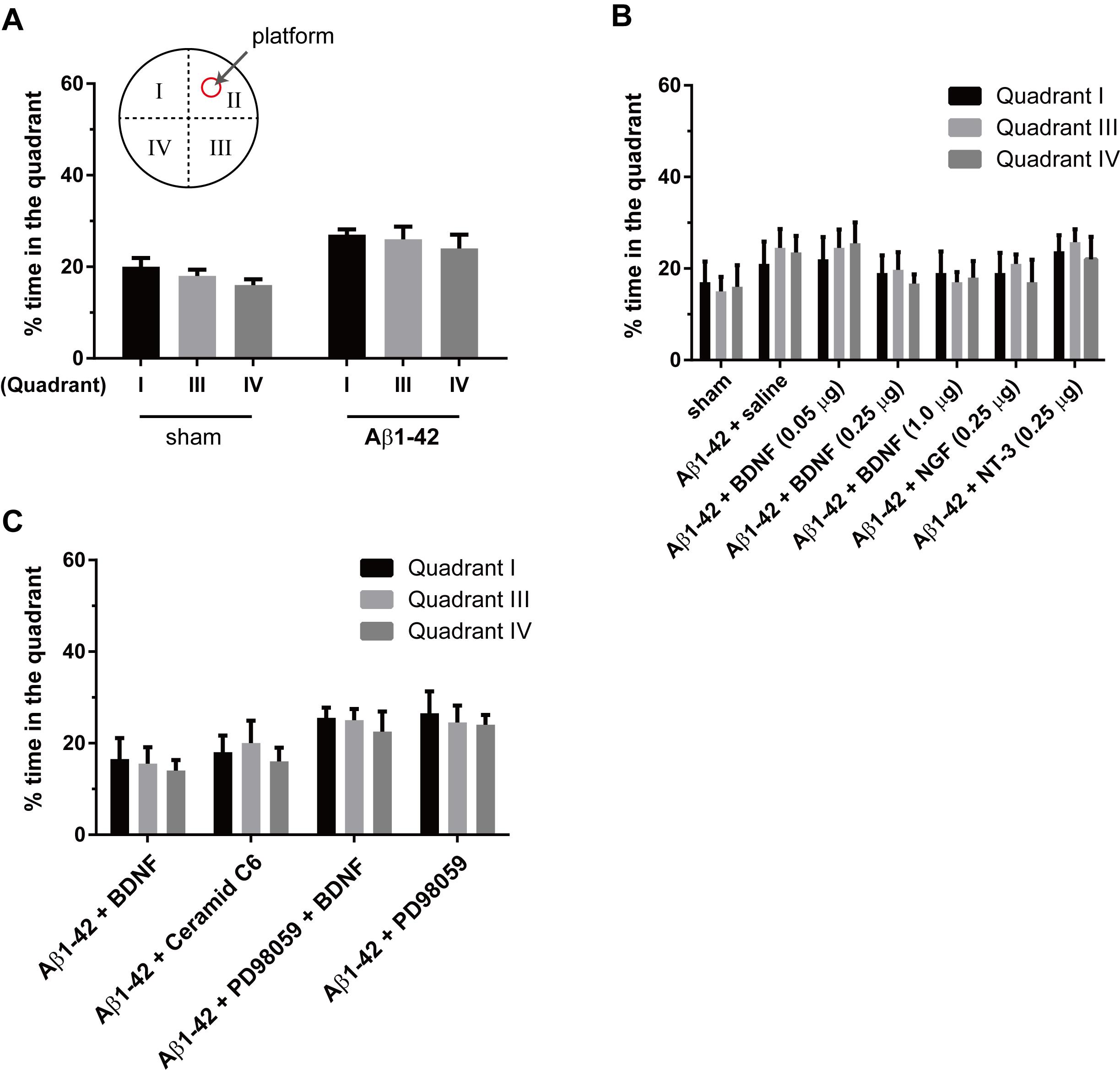

Supplement: S1 Fig — (A) Performance of rats in the Aβ1–42 and sham group. An schematic diagram showed the position for the four quadrants. (B) Performance of rats after intra-hippocampal injection with BDNF, NGF or NT-3. (C) Swimming time for the BDNF, Ceramid C6, PD98059 intra-hippocampal treated rats. (TIF) [file pone.0122415.s001.tif]
